# A Deep Learning Method for Human Sleeping Pose Estimation with Millimeter Wave Radar

**DOI:** 10.3390/s24185900

**Published:** 2024-09-11

**Authors:** Zisheng Li, Ken Chen, Yaoqin Xie

**Affiliations:** 1 Shenzhen lnstitute of Advanced Technology, Chinese Academy of Sciences, Shenzhen 518055, China; xjy109225@siat.ac.cn; 2 University of Chinese Academy of Sciences, Beijing 100190, China

**Keywords:** contactless sensing, deep learning, FMCW radar, sleep posture

## Abstract

Recognizing sleep posture is crucial for the monitoring of people with sleeping disorders. Existing contact-based systems might interfere with sleeping, while camera-based systems may raise privacy concerns. In contrast, radar-based sensors offer a promising solution with high penetration ability and the capability to detect vital bio-signals. This study propose a deep learning method for human sleep pose recognition from signals acquired from single-antenna Frequency-Modulated Continuous Wave (FMCW) radar device. To capture both frequency features and sequential features, we introduce ResTCN, an effective architecture combining Residual blocks and Temporal Convolution Network (TCN) to recognize different sleeping postures, from augmented statistical motion features of the radar time series. We rigorously evaluated our method with an experimentally acquired data set which contains sleeping radar sequences from 16 volunteers. We report a classification accuracy of 82.74% on average, which outperforms the state-of-the-art methods.

## 1. Introduction

Sleep is a critical component of human health. Sleep breath disorders like obstructive sleep apnea (OSA) are linked to various health issues, with a prevalence of 9% to 38%, increasing with age [1]. Sleep apnea patients may suffer from breath restarts numerous times every night when they sleep [2], which lead to a lack of work productivity, decreased cognitive function, car accidents, and daytime sleepiness [3,4]. Moreover, OSA is known as a risk factor for several complications including hypertension, diabetes, cardiovascular disease, and heart failure in untreated patients, even an increase sudden death, and the care of concomitant neurological diseases, such as epilepsy, stroke, multiple sclerosis, and headaches also becomes burdensome [5,6,7]. OSA has caused an economic burden of over 150 billion annually in the USA [8], including lost workplace productivity, increased healthcare utilization, motor vehicle collisions and workplace accidents and injuries.

Various clinical studies have demonstrated correlation relationships between OSA and sleep postures [9]. One group [10] suggests that adopting a supine posture could notably increase the risks of OSA due to the prolapse of the tongue and the soft palate against the pharyngeal wall due to gravity, while the prone position improves the apnea hypopnea index (AHI) and sleep quality. For patients with positional obstructive sleep apnea syndrome (OSAS), the supine posture will lead to significantly higher AHI [11]. Some studies [12,13] demonstrated that the OSA that occurred in lateral position is less severe than supine position.

Apart from OSA, sleep postures can also be observed as valuable markers of several disease, and significantly affect health. Keeping the supine posture for a longer period can be related to deterioration in the condition of Parkinson’s patients [14]. Keeping the same sleep posture for a long time can lead to pressure ulcers in the elderly and post-surgery patients [15,16]. Improper sleep postures can increase the risk of sudden death [17,18].

Therefore, implementing sleep posture monitoring is a crucial role in enhancing overall health and well-being. A variety of sensors have been developed to monitor sleep postures and behaviors, including body pressure sensors, cameras (including depth cameras) and wearable devices [19]. However, there are drawbacks in those existing solutions. Pressure mats, measuring the pressure intensity distribution, have been employed to characterize sleep postural behavior and estimate sleep quality [20]. Proper placement of a pressure mat is crucial for accurate measurement. The movements of patients can cause shifts in the position of body; therefore, repositioning is necessary. Different materials of the mattress can also affect the natures of the pressure profile [21]. Video recordings using red–green–blue (RGB) or RGB–depth images can capture and enable the observation of individuals’ sleep postures directly [22,23]. Camera-based methods are sensitive to the varying light conditions, the presence of blanket coverage [24,25,26,27,28] and privacy concerns may be raised [29]. Wearable devices utilizing actigraphy or accelerometry can measure physical activity and infer motion or behavioral activities [30]. Therefore, spectrogram analysis of data from wearable devices can be employed to estimate sleep postures based on body segment movements [31]. However, these systems or sensors may be costly or disrupt sleep, discouraging practical use.

In contrast, radar-based sensors do not encounter such dilemmas, and exhibit high penetration ability, which is capable of detecting human movements even through walls [32], and can be used for vital sign detection [33]. Numerous studies have explored the application of radar-based systems for the detection of human vital signs, including heart rate (HR), breathing rate, and breathing disorders [34,35,36,37]. There were also applications utilizing radar for the monitoring of body movements [38,39] and hand gestures [40,41]. The aforementioned applications suggest that the radar acquired data contains rich information to measure the macro and micro movement of human body, and it may be promising to distinguish the feature difference among different sleep postures from radar signals. A radar-based system has been proposed for sleep posture recognition [42], which exhibits an ability to capture both time and frequency features, including the movement and direction of sleep postural transition movement. Lai et al. proposed vision transformer-based deep learning methods to classify the sleep postures under multiple radar configurations [43]. The results showed that the dual-radar configuration (side + head) with the Transformer model could achieve the best sleep posture prediction. A new method for on-bed state detection was proposed by capitalizing on chest localization to monitor on-bed presence, posture, motion, and turning, providing a holistic view of sleep behavior [44]. As far as we know, the application of radar devices for sleep posture recognition has not been sufficiently studied, and is still a challenging problem.

In this study, we propose a deep learning method for sleep posture classification from radar sequences. The radar sequences are preprocessed, and we extract the statistical motion features from frequency domain to train the classification network. We propose a two-stage deep learning model ResTCN for sleep posture classification. We first use a residual block-based network structure as feature extractor, and use TCN as classifier to handle the sequential pattern.

## 2. Related Work

There have been several groups conducting research for sleep posture classification with radar signals. Fan et al. [45] recruited nine male and one female participant, and recorded 2 min for each posture per participant. Their primary conclusion was that the accuracy of the logic-based approach and the CNN-based method was of the same level, but the CNN-based approach only required the data in a window size below 30 s, and a sliding window size below 5 s, while the logic approach necessitated post-recording analysis of the entire sleep session; therefore, the CNN-based approach offered a distinct advantage in terms of real-time applicability. Yue et al. [46] proposed a system BodyCompass using FMCW radio equipped with an antenna array for sleep posture detection. They collected 224 nights of data from 26 subjects. They proposed a four-layer dense network to process the Filtered Multi-path Profile, and reported the accuracy outperforms the traditional machine learning methods significantly. Liu et al. [47] collected radar signal from 10 volunteers, 20 s for each posture, with basic scenario, quilt covered scenario and static reflector interference scenario. They compared the DNN-based methods with machine learning methods, and report the effectiveness of the DNN structure. They also reported that the presence of the quilt could change the extracted feature, and the results for scenarios with the presence of the static reflectors is acceptable. Kiriazi et al. [48] collected data from 20 subjects with 2.4 GHz and 5.8 GHz devices for 90 s for each posture and each device per person. They reported that the accuracy of posture classification with dual frequency data set outperforms both single frequency data sets. Lai et al. [49] recruited 18 healthy young adults (12 males and 6 females) in their study, and collected 720 samples (18 participants × 4 postures × 10 repetitions). Each posture was recorded for 20 s. They evaluated traditional machine learning methods and deep learning methods, with different radar combinations, and concluded that the accuracy of the dual radar was significantly better than single radar. Lai et al. [43] further collected data with radar devices from three different positions. They recruited 30 volunteers and collected 1200 samples, each for 15 s. They first proposed to use Vision Transformer network structure for the posture classification problem. The Swin-transformer V2 structure with the head and side radar combination showed best accuracy. Lai et al. [50] also conducted experiment by recruiting 70 adults to collect data from devices with different position and orientation, and compared the performance of different deep networks under different combination of signals. Yao et al. [51] recruited 17 subjects and collected 1400 samples of 5 s for four postures. They proposed a encoder–decoder structure.

From the literature review, we can summarize some common points of the sleep posture classification experiments. The participants usually do not achieve sleep during experiments; instead, they are instructed to lie in different postures in a given order, and are required to stay stationary during data collection. The exclusion criterion of the participants are usually in common, including physical disability, obesity, pregnancy, or any cardio respiratory problems, and difficulties in maintaining or switching specific postures in bed. In most of the experiments, the number of volunteers varies from 10 to 20, which can satisfy the generalization requirement of data set for deep network training. The scales of data sets do not follow a common standard. If the time of data collection process is too short, the scale of the data set will not be sufficient to achieve satisfactory accuracy. On the other hand, a too long recording time can affect the comfort of the volunteers, making it difficult for the subjects to stay stationary during the data collection process. Therefore, most of the state-of-the-art experiments recorded several minutes for one posture per person.

We can see that, in the current state-of-the-art studies, some groups applied existing technologies directly to the radar collected data for sleep posture classification, such as the traditional machine learning methods like regression, SVM, Random Forest, etc. [49], and the existing deep learning models, like transformer-based network structures [43,49,50]. Some groups proposed specifically designed networks, which were based on convolutions [45,47,51] or full connections [46] only, but not utilizing the temporal information. However, the radar device captures time sequences, which contain important feature patterns for different sleep postures. Therefore, in this study, we propose a novel two-stage network structure by combining the convolution-based backbone ResNet for feature extraction and the Temporal Convolution Network (TCN) to capture long-time dependencies and sequential patterns in radar time series. We will show that the combination of the ResNet and the TCN will better recognize the features from the radar data, by achieving better accuracy over the state-of-the-art comparative methods.

Also, current methods usually tried to extract the information directly from the radar acquired data, while in this study, we propose a novel form of feature, the statistical motion features derived from the range-FFT images, to capture the micro movement of human body, such as respiration and heart-beats. We will show that the proposed statistical motion features can enhance the feature representation compared to the traditional radar data features, which essentially improve the accuracy.

Therefore, we summarize the main contributions of this manuscript:

1. We propose a novel two-stage deep learning model ResTCN for sleep posture classification with radar data. We first use ResNet as feature extractor, and use TCN as classifier to handle the sequential pattern.

2. We propose a novel form of feature, the statistical motion features to enhance the feature representation of the radar data.

## 3. Method

### 3.1. System Setup and Data Acquisition

**Figure 1:**
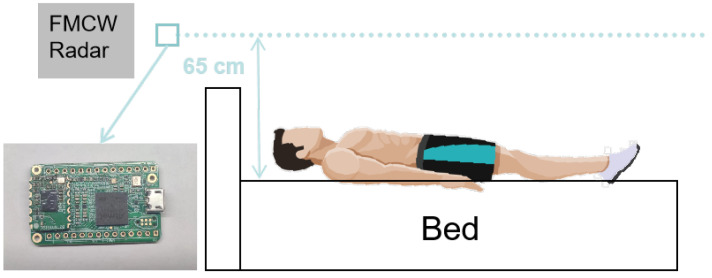
Experimental bedroom environment with a FMCW radar devices mounted on the wall.

The data used in this study is an experimentally acquired radar sequence set in our lab with an Infineon BGT60TR13C FMCW radar system. The working parameter of the radar is shown in Table 1. The radar system is mounted on the wall, positioned 0.65 m above the bed, with a pitch angle tilted approximately 45 degrees downward toward the bed to cover the interested part of the torso, as shown in Figure 1.

In this data set, we recruited 16 participants to acquire the radar sequences of four different sleep postures. Their average age was 30 (SD: 5.31, range 23–38). The mean weight and height were 172 cm (SD: 5.99 cm, range 160–180 cm) and 67.3 kg (SD: 9.83 kg, range 53–90 kg), respectively. The exclusion criteria included physical disability, obesity, pregnancy, or any cardio-respiratory problems, in addition to participants with difficulties in maintaining or switching specific postures in bed.

**Figure 2:**
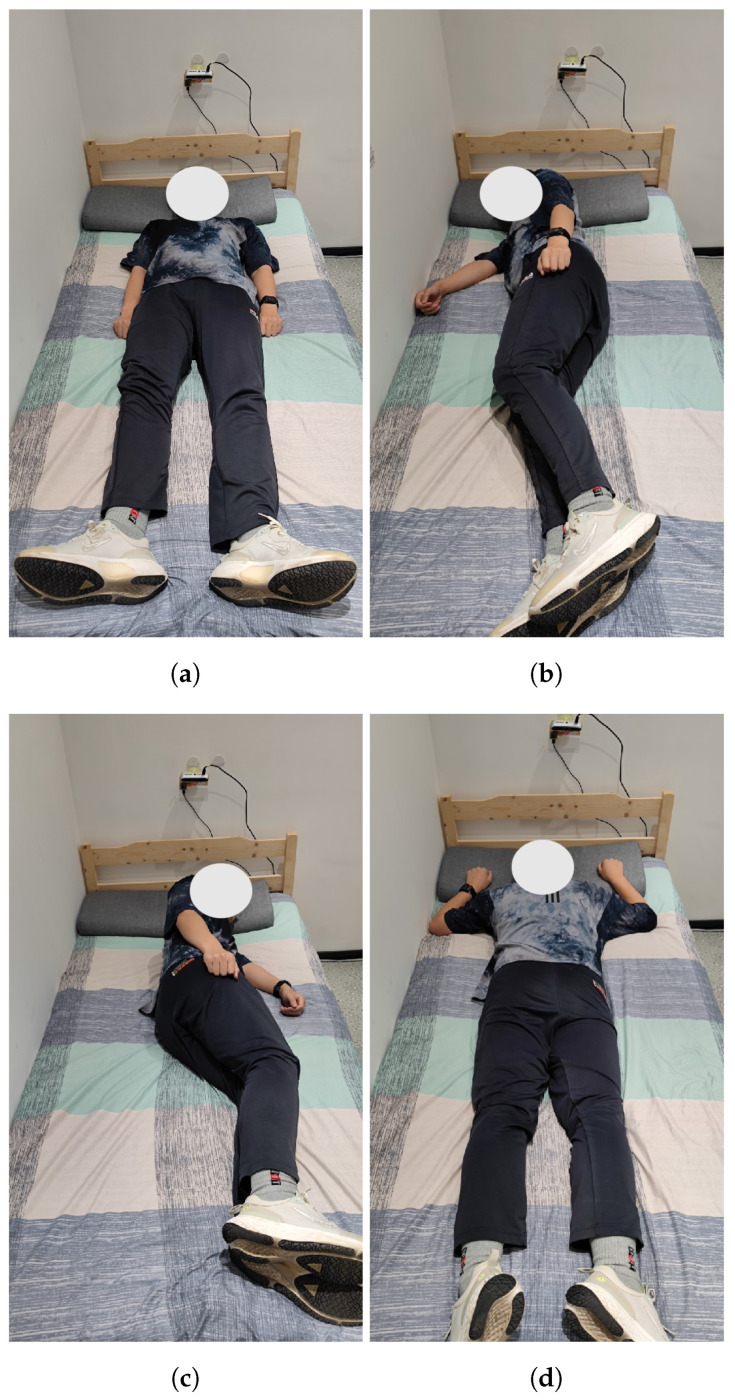
The four postures: (**a**) supine, (**b**) left-side lying, (**c**) right-side lying, and (**d**) prone.

The participants were commenced to lie on the middle of the bed, supported by a pillow. Then, they were guided to lie in various stationary postures, as shown in Figure 2, in the specified order: (1) supine, (2) right lateral, (3) left lateral, and (4) prone, each for 10 min for data acquisition. The overall acquisition time is 640 min (16 participants × 4 postures × 10 min). In this study, we mainly focus on the recognition of the feature differences of 3 gesture categories: supine, lateral and prone; therefore, we combine the left and right lateral data into one category. Compared with the literature reviewed in the Related Work section, we can see that the design of the experiments in this study does not contradict with the principles of the existing state-of-the-art works.

Informed consent was obtained from all the volunteers. The experimental procedure for data acquisition and utilization in this research has been approved by the ethics committee of Shenzhen Institute of Advanced Technology, Chinese Academy of Sciences. The committee reference number is SIAT-IRB-240615-H0890.

### 3.2. Data Processing

#### 3.2.1. Preprocessing

Frequency Modulated Continuous Wave (FMCW) is a specialized millimeter-wave technology. The transmitting (TX) antenna of the FMCW radar transmits a signal called a “chirp”, which is a sinusoid whose frequency increases lineally with time. Once the chirp arrives at the focused object, the signal will be reflected and received at the receiving (RX) antenna. The RX signal and TX signal are mixed into a resulting signal, “IF signal”. For an object at a distance D from the radar, the discrete IF signal $X_{IF}$ can be represented as

(1) $$X_{IF}\left(m\right)=A\sin\left(2\pi f_{0}T_{s}m+\phi_{0}\right),\;m=1,2,\ldots M$$

where $X_{IF}$ is the mixed signal, $f_{0}=\frac{s2D}{c}$, and $\phi_{0}=\frac{4\pi D}{\lambda }$. D is the distance between object and radar, s is the slope of chirp, and λ is the wavelength of radar, $T_{s}$ is the sampling interval, and *M* is the number of fast time indices. We accumulate the discrete IF signal of each chirp over time to form a M×N matrix $R_{MN}$, where $R_{MN}=\left[x_{1},x_{2},\ldots ,x_{N}\right]$, and $x_{i}={\left[x_{IF}^{i}\left(1\right),x_{IF}^{i}\left(2\right),\ldots ,x_{IF}^{i}\left(M\right)\right]}^{T}$ is the IF signal of chirp with index *i*. In our case, the slow time index *N* is 640 (chirp rate×frame rate) for 1 s, the fast time index M=128 is the sampling number of the IF signal of each chirp.

The raw radar signal $R_{mn}$ contains a DC component, and also static clutter representing uninterested objects and obstacles in the environment. To mitigate the impact of the DC noise and static clutter, the mean subtraction method is applied to the raw radar signal in two consecutive steps. The DC suppression can be expressed by Equation (2), and the static clutter suppression can be expressed by Equation (3).

(2) $${\bar{R}}_{mn}=R_{mn}-\frac{1}{N}\sum_{i=0}^{N-1}R_{mi}$$

(3) $$Y_{mn}={\bar{R}}_{mn}-\frac{1}{M}\sum_{i=0}^{M-1}{\bar{R}}_{in}$$

#### 3.2.2. Range Fourier Transform (Range-FFT)

We consider each chirp as a quasi-static scenario, where the relative velocity within one chirp is 0, and the relative distance R remains constant. We apply Fourier Transform to each chirp, and obtain the spectrum where each peak represents an obstacle. This process is defined as the Range Fourier Transform (range-FFT). The spectra are stored in a K × N array, denoted as F, where

(4) $$F_{kn}=\sum_{m=0}^{M-1}Y_{mn}e^{\frac{-j2\pi mk}{M}},\;k=1,2,\ldots K,\;n=1,2,\ldots N$$

where K is the frequency index, and N is the slow time index. In our case, K equals to 64, and N equals to 640 for 1 s.

#### 3.2.3. Statistical Motion Features

In order to capture the micro movement of human body, such as respiration and heartbeats, we propose to extract the statistical motion features from the range-FFT spectrogram to train the model. A sliding window with a width of W along the slow time indexes is applied to the spectrogram, with a step of S. For an arbitrary window with index i, the standard deviation of the spectrogram for an arbitrary range bin with index k is calculated as

(5) $${std}_{k,i}=\sqrt{\frac{1}{W}\sum_{t=i\times S}^{i\times S+W-1}{\left(F_{kt}-\bar{F_{ki}}\right)}^{2}}$$

(6) $$\bar{F_{ki}}=\frac{1}{W}\sum_{t=i\times S}^{i\times S+W-1}F_{kt},\;k=1,2,\ldots K,\;i=1,2,\ldots N_{W}$$

where K is the frequency index, and N_W_ is the total number of sliding windows. In our case, K = 64, and we choose W to be 320 (10 frames × 32 chirps), which is 0.5 s, S to be 64 (2 frames × 32 chirps), which is 0.1 s. The sliding window covers a slow time index range of 6 s, which means $N_{W}$ = 60 in our case.

To cover only the interested regions of the human body, we select the data inside a bin window with a size of K_1_ × N_W_. The range resolution of our radar device is 4 cm; therefore, we optimally choose $K_{1}=40$, which covers a range of 1.6 m. The optimal position of the bin window is decided by maximize the sum of the standard deviations within the bin window.

**Figure 3:**
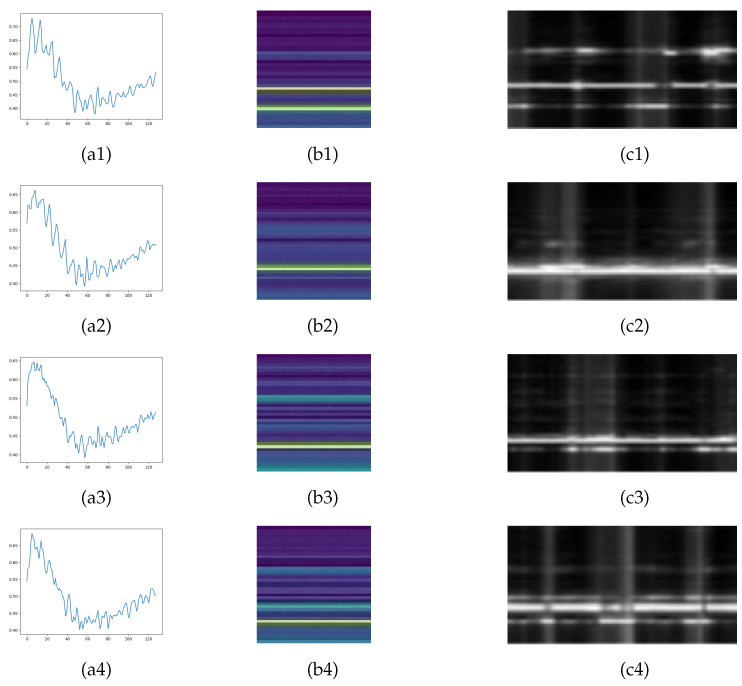
Typical signal examples of different postures. (**a1**–**a4**) Typical chirp time sequences of 4 postures: supine, left-side lying, right-side lying, and prone. (**b1**–**b4**) Range-FFT images of the 4 postures (1 frame, 32 chirps). (**c1**–**c4**) Statistical motion feature images of the 4 postures (6 s).

The standard deviations within the optimal bin window are accumulated to form a K_1_ × N_W_ feature image X, where $X_{k,j}={\mathrm{std}}_{k,j}$, $K_{1}=40$, and $N_{W}$ = 60 for our case. Typical examples of the statistical motion feature images of different sleep postures are shown in Figure 3.

### 3.3. Data Augmentation

In order to improve the generality of the model, data augmentation techniques, including time shift, range shift, and mix-up were applied.

For time shift process, each feature image was moved along the slow time index by a random offset ranged in [−1, −0.5] or [0.5, 1] seconds. The time shift reduces the effect of data truncation, and improves the coverage of complete respiratory cycles. For range shift process, each feature image was moved along the range bin index by a random offset ranged in [−10, −5] or [5, 10]. The range shift aid the model to reduce the effect of the lying position difference.

**Figure 4:**
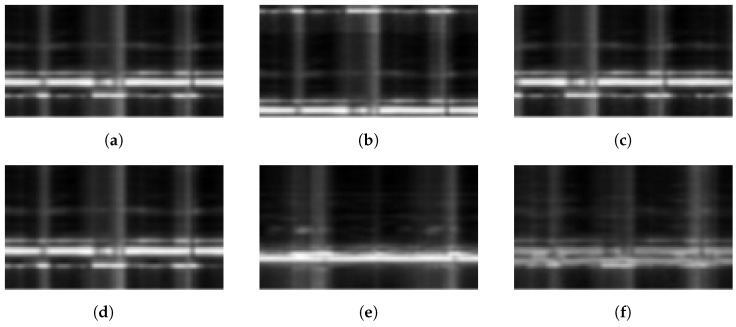
Typical examples of the augmented samples. (**a**) Original: sample from the original feature image set. (**b**) Range shift: range shifted image with an offset of 10 pixels. (**c**) Time shift: time shifted image with an offset of 1 s. (**d**,**e**) Feature image 1 of prone, feature image 2 of left side lateral: feature images of supine and left side lateral. (**f**) Mix-up of feature image 1 and 2 (λ = 0.5): mix-up image of (**d**,**e**) with λ = 0.5.

The mix-up technique [52] was introduced to improve the generalization of empirical risk minimization (ERM). Two feature images from different categories and the corresponding labels were linearly combined to generate a virtual feature image, based on a random weight picked according to the beta distribution, as Formula (7), where λ∈Beta(α,α) and α<1. Typical examples of the augmented samples are shown in Figure 4.

(7) $$\begin{array}{cc} \; & \bar{X}=\lambda x_{i}+\left(1-\lambda \right)x_{j}\\ & \bar{Y}=\lambda y_{i}+\left(1-\lambda \right)y_{j} \end{array}$$

### 3.4. ResTCN

**Figure 5:**
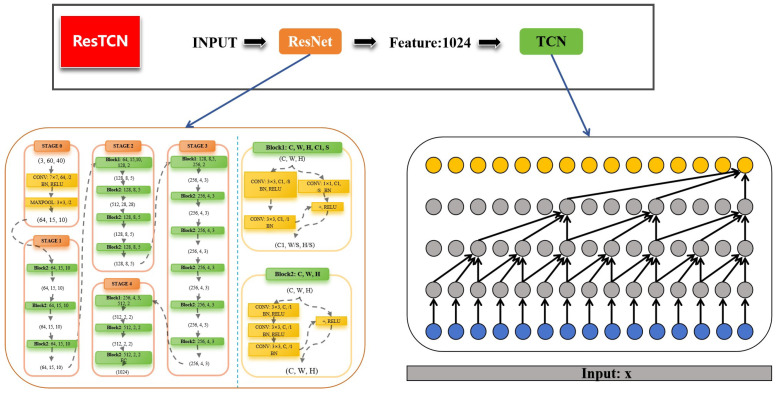
Architecture of ResTCN.

In this section, we will describe the proposed scheme for sleep posture recognition in detail. The overall process of the scheme is shown in Figure 5. We first used a backbone network based on residual blocks to extract meaningful features from the augmented feature set obtained in Section 3.3. The extracted features were feed into the Classification Network for sleep posture recognition.

We proposed to use the ResNet [53] network as the Feature Extraction Network. With its skip connections and residual blocks, ResNet allowed the training of deeper network structures, and showed hierarchical feature extraction capability [54]. ResNet has demonstrated superior performance in capturing complex features and patterns in various image recognition tasks [55,56,57]. The structure and parameters of the network in this work are shown in Figure 5 in detail. The feature image is cloned to the three channels of the input image. A feature vector with a length of 1024 is obtained for classification.

We proposed to use Temporal Convolution Network (TCN) [58] as the Classification Network. TCN is well-suited for tasks requiring temporal modeling due to its parallelizable dilated convolutions and global receptive field, and shows promising results in capturing long-time dependencies and sequential patterns in time series data [59,60]. The detail structure of the network applied in our case is shown in Figure 5.

The proposed deep learning method was implemented with PyTorch1.7 on a NVIDIA 4090 GPU device. We chose the AdamW optimizer to minimize the cross entropy as Equation (8). The initial learning rate is set to 0.005, with a L2 regularization coefficient of 0.001. The learning rate was scaled down 10 times every 10 training epochs. The model was trained for 100 epochs.

(8) $$J_{po}\left(\theta \right)=-\frac{1}{M}\sum_{i=0}^{M}y_{p}^{\left(i\right)}log{\hat{y}}_{p}^{\left(i\right)}\left(\theta \right)$$

## 4. Results

In this section, we evaluate the performance of the proposed sleep posture classification scheme, and compare the proposed method with state-of-the-art methods.

### Sleep Posture Classification Results

To test the performance of the proposed method, We randomly chose six volunteers as the test set. From the remaining 10 volunteers, samples of eight randomly chosen volunteers were used to train the network, while the remaining two volunteers were used as the validation set. We used the following metrics to evaluate the model accuracy performance:

(9) $$\begin{array}{cc} \; & \mathrm{Accuracy}=\frac{TP+TN}{TP+TN+FP+FN} \end{array}$$

(10) $$\begin{array}{cc} \; & \mathrm{Precision}=\frac{TP}{TP+FP} \end{array}$$

(11) $$\begin{array}{cc} \; & \mathrm{Recall}=\frac{TP}{TP+FN} \end{array}$$

(12) $$\begin{array}{cc} \; & F1=\frac{2\times \mathrm{Precision}\times \mathrm{Recall}}{\mathrm{Precision}+\mathrm{Recall}} \end{array}$$

We compared our proposed method with the following state-of-the-art methods:

1. We applied the SVM-based method [61] to our augmented feature image set. The features of the image are extracted by PCA, and the classification is achieved by SVM with Radial Basis Function (RBF) kernel. Grid-search cross-validation is applied to find the best parameters of SVM. The search sets of the parameters of the SVM, C and gamma are [0.01, 0.1, 1, 10, 100] and [0.01, 0.1, 1, 10, 100], respectively.

2. ShuffleNet [62] is a convolutional neural network architecture widely employed for classification tasks. ShuffleNet features a unique design comprising shuffle units, which facilitate efficient information exchange across layers. It is characterized by its lightweight structure, making it suitable for deployment on resource-constrained devices [63].

3. DenseNet [64], a distinctive convolutional neural network architecture, introduces a novel connectivity pattern that fosters extensive information exchange among network layers. Unlike traditional architectures, where each layer is connected only to its subsequent layer, DenseNet incorporates dense connections, enabling direct communication between all layers within a block. This dense connectivity facilitates feature reuse, and enhances gradient flow during training, effectively addressing the vanishing gradient problem. By encouraging feature propagation and fostering feature reuse, DenseNet achieves remarkable parameter efficiency while maintaining high model accuracy. With its unique design, DenseNet has emerged as a powerful tool for various computer vision tasks, demonstrating superior performance in image classification, object detection, and semantic segmentation [65,66].

4. Vision transformer (VIT) [67] is a groundbreaking deep learning model designed specifically for image classification tasks. Unlike CNN, VIT employs a self-attention mechanism to capture global information within data; therefore, it has achieved promising results in various fields [68,69]. In our case, we use VIT to analyze the the pattern of the statistical feature images of the spectrogram.

5. Swin transformer V2 [70] is an advanced deep learning model that builds upon the traditional Transformer architecture by introducing a hierarchical feature representation and shifted windows, enabling it to capture both local and global context effectively. Unlike VIT, which processes images as a whole, Swin Transformer V2 divides the image into non-overlapping windows and performs self-attention within each window, followed by shifting the windows to capture cross-window connections. Swin transformer V2 and its variants have received growing interest in image processing realms. In a recent study, Lai et al. [43] first proposed to use the vision transformer network for sleep posture classification with radar image features. They reported that the Swin transformer V2 structure with the head and side radar combination showed best accuracy. For the head radar scenario, which is the case of this manuscript, the Swin transformer V2 also outperforms the comparative methods.

**Figure 6:**
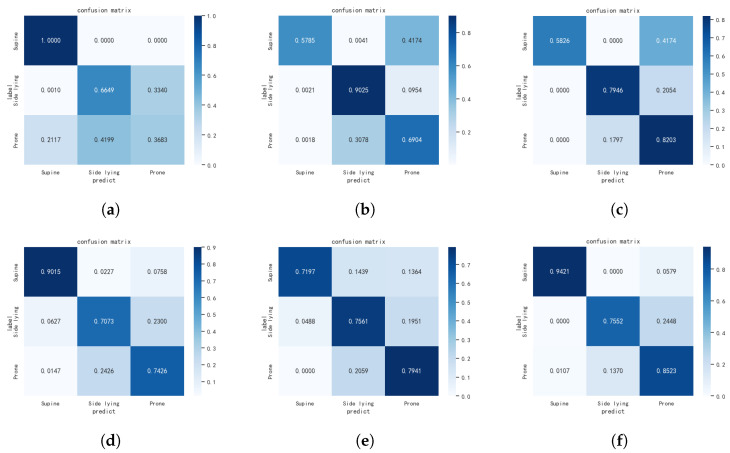
The confusion matrix of the proposed method and relative SOTA methods. (**a**) SVM. (**b**) ShuffleNet. (**c**) DenseNet. (**d**) Vit. (**e**) Swin Transformer V2. (**f**) ResTCN (proposed).

All of the comparative methods were trained and evaluated under the same hardware and software platform configurations of the proposed method, with the same data processing and augmentation strategies. The comparison of accuracy performance is summarized in Table 2, and the typical confusion matrices are depicted in Figure 6. We can see that the proposed method ResTCN outperforms all other comparative methods in terms of accuracy, F1-score, Precision, and Recall. The combination of residual blocks and TCN allows the proposed network to recognize both the spatial patterns and the sequential dependencies, resulting in superior classification accuracy over the convolution-based comparative methods. SVM is a powerful method for certain classification tasks, but in our case, the ability to capture the frequency domain features is limited. VIT and Swin transformer V2 may be powerful in natural image processing, but it requires a very large scale of data for training to obtain optimal feature recognition abilities.

## 5. Discussion

### 5.1. Ablation Experiment

#### 5.1.1. Model Architecture

In this section, we show the necessity of the combination of the residual blocks and TCN structures. We conducted a comparison experiment by applying only ResNet and TCN, respectively. When applying the ResNet, the augmented statistical motion feature images are fed into the network, and a FC layer is used for classification. When applying TCN, the feature images are flatten, and the output of the last time index is connected to a FC layer for classification. We conducted three experiments, namely ShuffleNet + TCN, DenseNet + TCN, and ResNet + LSTM for performance comparison among different combinations of backbone network structures. The results are summarized in Table 3; we can see that the ResTCN model consistently outperforms the other combinations across all evaluation metrics, including accuracy, precision, recall, and F1 score. Therefore, we can conclude that the proposed ResTCN structure show better performance. The fusion of the residual blocks and TCN structure can better recognize the features from the spectrogram-based images.

#### 5.1.2. Statistical Motion Feature Extraction

We conducted ablation experiments to evaluate the effect of the motion feature extraction in our proposed method by training and testing the proposed network with the range-FFT images and the motion feature images. The results are detailed in Table 4. Notable improvement in classification performance is observed, highlighting the crucial role of statistical motion features in capturing the difference of micro human movement patterns among different sleep postures.

#### 5.1.3. Data Augmentation

We compare the classification results with and without data augmentation. The results are summarized in Table 5. We can see that with the data augmentation the performance is improved. This improvement demonstrates the effectiveness of data augmentation in enriching the diversity of the training data set and improving the generalization of the model.

### 5.2. Parameter Selection

To determine the most suitable window size along the range axis, we conducted an investigation to compare the classification accuracy metrics under different window size. The range of window size values explored in this experiment included 30, 40 (optimal), 50, and 60 range bins. The comparison details are shown in Table 6. Compared to other window size, 40 range bins ensures a more comprehensive coverage of the interested part of the human body for respiratory feature extraction, and can effectively mitigates radar multi-path effects, minimizing the environmental impact on features.

We also study the choice of range covered by the sliding window along the slow time axis. The range of sliding window included 2 s, 6 s, and 10 s. The comparison details are shown in Table 7. Compared to the 2 s sliding window, the 6 s window shows significant improvements in all accuracy metrics, while the improvement of accuracy metrics from 6 s to 10 s is minor with greater computation load and calculation time. A smaller window size along the slow time axis also reduces the time delay for detection of posture change. Therefore, we can conclude that the 6 s window size is the optimal choice, which achieves the balance of accuracy and efficiency.

### 5.3. Limitations

There are several limitations in this study. The scale of the data set is not sufficiently large, which keeps the vision transformer showing superior ability in image classification tasks. In our future work, we will further collect data to fine tune the vision transformer network to recognize the features among different sleep postures.

Also, in the data we acquired in this study, we currently only include the data from healthy subjects, but not the data from subjects with OSA. Identifying OSA is a labor-intensive task, and usually requires specialized facilities and experts with qualifications. In our future work, we will work with our collaborative facilities for further data acquisition and analysis from OSA patients. Also, we will include cases with the presence of blanket coverage in the future work.

## 6. Conclusions

This study proposed the ResTCN, an effective architecture that leverages ResNet and TCN backbone structures to classify various human sleep postures acquired from FMCW radar device. The statistical motion features are extracted from range-FFT spectrograms, and the data augmentation techniques are introduced to address the over-fitting issue. We rigorously test the proposed network with an experimentally acquired data set. The classification results for the three sleep postures show promising accuracy, and surpass the state-of-the-art methods. In conclusion, the proposed method is promising in the application of non-contact human sleep monitoring.

## Figures and Tables

**Table 1:** The parameters of the radar.

Parameters	Values
Bandwidth	5.0 GHz
Start frequency	58 GHz
Chirp duration	133 µs
Chirp repetition time	463 µs
No. samples per chirp	128
Frame rate	20 Hz
ADC sampling rate	1 MHz
Range resolution	3 cm
Velocity resolution	1.34 m/s

**Table 2:** The accuracy comparison of the proposed method and comparative methods.

Algorithm	Accuracy	Precision	Recall	F1Score
SVM [61]	0.6627	0.6412	0.6778	0.6552
ShuffleNet [62]	0.7652	0.7970	0.7238	0.7400
DenseNet [64]	0.7507	0.8063	0.7325	0.7403
Vit [67]	0.7621	0.7587	0.7838	0.7652
Swin transformer V2 [70]	0.7567	0.7623	0.7566	0.7517
**ResTCN (proposed)**	**0.8274**	**0.8453**	**0.8499**	**0.8404**

**Table 3:** The accuracy comparison of ResNet, TCN, and different combinations of network structures.

Algorithm	Accuracy	Precision	Recall	F1Score
**ResTCN (proposed)**	**0.8274**	**0.8453**	**0.8499**	**0.8404**
ResNet	0.7980	0.8103	0.7960	0.8021
TCN	0.7452	0.7856	0.7230	0.7343
ShuffleNet+TCN	0.7766	0.8091	0.8019	0.7950
DenseNet+TCN	0.7736	0.8192	0.8225	0.7995
ResNet+LSTM	0.7592	0.7834	0.7904	0.7822

**Table 4:** The accuracy comparison of data with/without the motion feature extraction.

Method	Accuracy	Precision	Recall	F1Score
**Motion feature image**	**0.8274**	**0.8453**	**0.8499**	**0.8404**
Original range-FFT image	0.6852	0.7018	0.6633	0.6780

**Table 5:** The accuracy comparison of different combinations of data augmentation options.

Method	Accuracy	Precision	Recall	F1Score
**Proposed**	**0.8274**	**0.8453**	**0.8499**	**0.8404**
Original	0.7761	0.7946	0.7688	0.7784
Mixup (-)	0.7975	0.8336	0.8163	0.8108
Time shift (-)	0.8129	0.8201	0.8282	0.8241
Range shift (-)	0.8044	0.8084	0.8234	0.8227

**Table 6:** The accuracy comparison of different range window size.

Window Size	Accuracy	Precision	Recall	F1Score
30	0.7947	0.8222	0.7902	0.7955
40 (optimal)	0.8274	0.8453	0.8499	0.8404
50	0.8194	0.8234	0.8234	0.8276
60	0.8123	0.8320	0.8189	0.8241

**Table 7:** The accuracy comparison of different slow time window sizes.

Window Size	Accuracy	Precision	Recall	F1Score
2 s	0.7673	0.8067	0.7398	0.7448
6 s (optimal)	0.8274	0.8453	0.8499	0.8404
10 s	0.8354	0.8520	0.8202	0.8375

## Data Availability

Data are available upon request.
